# AlleleAnalyzer: a tool for personalized and allele-specific sgRNA design

**DOI:** 10.1186/s13059-019-1783-3

**Published:** 2019-08-15

**Authors:** Kathleen C. Keough, Svetlana Lyalina, Michael P. Olvera, Sean Whalen, Bruce R. Conklin, Katherine S. Pollard

**Affiliations:** 1 Pharmaceutical Sciences and Pharmacogenomics Graduate Program, University of California, San Francisco, CA USA; 2 Gladstone Institutes, San Francisco, CA USA; 3 Bioinformatics Graduate Program, University of California, San Francisco, CA USA; 4 Departments of Biostatistics, Medicine, Ophthalmology and Pharmacology, University of California, San Francisco, CA USA; 5 Department of Epidemiology & Biostatistics, Institute for Human Genetics, Quantitative Biology Institute, and Institute for Computational Health Sciences, University of California, San Francisco, CA USA; 6 Chan Zuckerberg Biohub, San Francisco, California USA

**Keywords:** CRISPR, sgRNA design, Genomics, Genome surgery, Genome editing, Computational biology

## Abstract

The CRISPR/Cas system is a highly specific genome editing tool capable of distinguishing alleles differing by even a single base pair. Target sites might carry genetic variations that are not distinguishable by sgRNA designing tools based on one reference genome. AlleleAnalyzer is an open-source software that incorporates single-nucleotide variants and short insertions and deletions to design sgRNAs for precisely editing 1 or multiple haplotypes of a sequenced genome, currently supporting 11 Cas proteins. It also leverages patterns of shared genetic variation to optimize sgRNA design for different human populations. AlleleAnalyzer is available at https://github.com/keoughkath/AlleleAnalyzer.

**Electronic supplementary material:**

The online version of this article (10.1186/s13059-019-1783-3) contains supplementary material, which is available to authorized users.

## Background

CRISPR genome editing’s success depends on the efficiency and specificity of the guide RNA (sgRNA) design. Current sgRNA design tools primarily predict the efficiency and specificity of sgRNAs using features such as prevalence of off-target sites, epigenetic marks, and chromatin accessibility [1–3]. Generally, sgRNAs are designed using reference genomes, such as the hg38 assembly for human or the GRCm38 assembly for mouse. However, these sgRNAs are used on cell lines or organisms with many nucleotide differences from the reference (e.g., on average 0.1% of a human genome [4]). While sgRNAs can sometimes tolerate a single base pair mismatch, frequently, these mismatches negatively impact sgRNA efficiency and render imprecise results of specificity prediction [5, 6], with potentially serious effects when sgRNAs are deployed.

Previous work analyzing data from ExAc and the 1000 Genomes Project determined that genetic variants could have a large impact on sgRNA efficiency and specificity, demonstrating the need for a tool to design sgRNAs using genetic variation and to identify sgRNAs that could work in many people to facilitate regulatory approval for therapeutic use [6, 7]. The solution implemented in this previous work aimed to avoid the negative effects of genetic variation by identifying universal sgRNAs located in the sites with little to no genetic variation and possessing few predicted off-targets [6]. However, many loci one may wish to edit lack variation-free regions for designing such sgRNAs (see below). We propose personalized sgRNA design, which uses the genetic variants in a genome or population, as a second approach that offers more flexibility in sgRNA design. We further note that genetic variation is not only a challenge for sgRNA design, but also an opportunity. Specifically, the use of CRISPR in research areas such as haploinsufficiency, genomic imprinting, and dominant negative diseases requires allele-specific sgRNA design, which may be accomplished using heterozygous variants.

To address these needs, we developed AlleleAnalyzer, an open-source Python software tool that designs personalized and allele-specific sgRNAs for individual genomes, identifies pairs of sgRNAs to generate excisions likely to block the expression of a gene, and leverages patterns of shared genetic variation across thousands of publicly available genomes to design sgRNA pairs that will have the greatest utility in a target population.

## Results and discussion

**Fig. 1:**
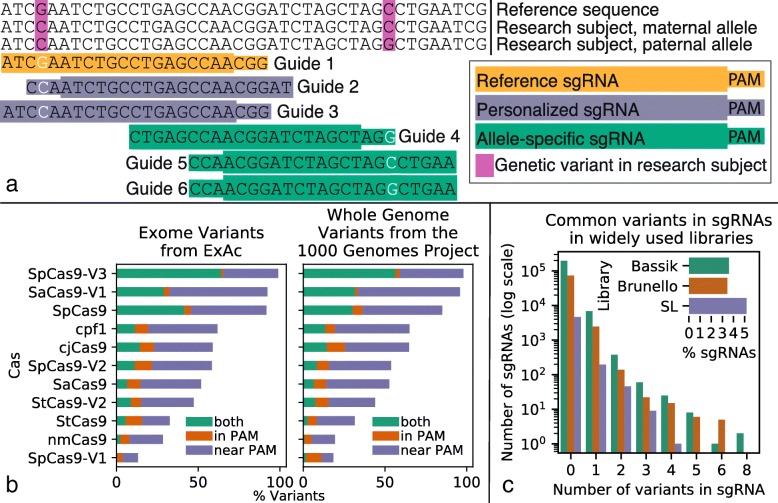
Analysis of allele-specific sgRNA sites. **a** In a sample genome, tools designing sgRNAs for the reference genome are imperfect matches due to genetic variants, exemplified by guide 1. AlleleAnalyzer designs personalized sgRNAs, as demonstrated by guides 2 and 3, which incorporate homozygous and avoid heterozygous variants, thus designing a guide perfectly matched to both alleles in a subject. It also designs personalized allele-specific sgRNAs based on the incorporation of heterozygous variants, shown by guides 4–6. Guides 4 and 6 target the paternal allele, while guide 5 targets the maternal allele. **b** Most variants annotated by the 1000 Genomes Project (1KGP) and the Exome Aggregation Consortium (ExAc) are in or near a PAM site. **c** Analysis of common variants (minor allele frequency (MAF) greater than 5% in 1KGP) and all variants in an individual cell line (WTC) within commonly used sgRNA libraries

Incorporating genetic variation into sgRNA design enables personalized and allele-specific CRISPR experiments. We define a personalized sgRNA as an sgRNA designed to incorporate the genetic variants of the research subject. A genetic variant can impact sgRNA sites by being located in or near a protospacer adjacent motif (PAM site), potentially generating or eliminating sgRNA sites in an individual in a heterozygous or homozygous manner. Beyond being an impediment to designing effective sgRNAs, these variants enable the design of personalized, non-allele-specific sgRNAs (incorporating homozygous variants and avoiding heterozygous variants to match both alleles) and allele-specific sgRNAs (incorporating heterozygous variants). The way in which genetic variation impacts or is incorporated into sgRNA design depends on the use case for the sgRNA and variant zygosity (Fig. 1a).

**Fig. 2:**
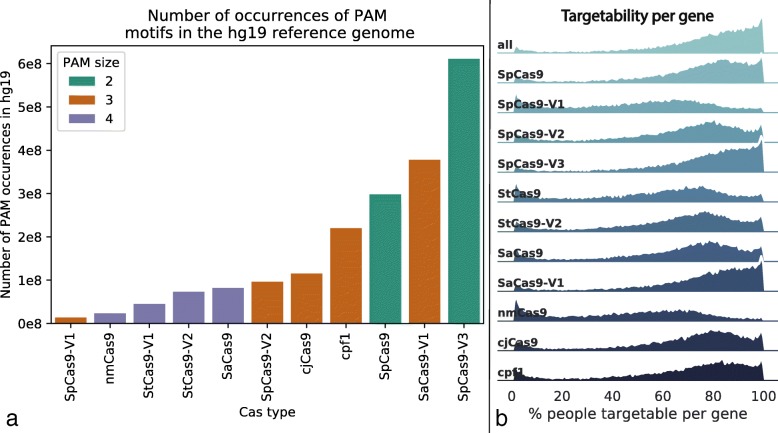
Target availability by Cas enzyme. **a** PAM frequencies in the human reference genome hg19, colored by the size of the PAM site (number of non-“N” nucleotides in motif). **b** In this faceted density plot, the height of the colored portion indicates the proportion of genes where the specified percentage (on the *x*-axis) of the 1000 Genomes cohort is putatively targetable

Because Cas nucleases have different PAM sequences, a variant may impact an sgRNA site for one Cas but not another. We analyzed 11 Cas types (Additional file 1: Table S1), genome-wide variants from > 2500 individuals from the 1000 Genomes Project [8] (1KGP), and exome variants from > 60,000 individuals in the Exome Aggregation Consortium (ExAc). From these analyses, we discovered that most variants impact sgRNA sites for at least one Cas type, even when considering only the variants in PAMs, which are putatively more allele-specific [9] (Fig. 1b). The likelihood that a variant impacts an sgRNA site differs across Cas nucleases (1KGP: range 19–98%; ExAc: range 13–99%), is positively correlated with PAM frequency in the reference genome (1KGP: Pearson rho = 0.89, *p* = 0.0002; ExAc: Pearson rho = 0.84, *p* = 0.0011, Fig. 2a), and is negatively correlated with PAM size (1KGP: Pearson rho = − 0.71, *p* = 0.014; ExAc: Pearson rho = − 0.74, *p* = 0.0094). In fact, > 3% of sgRNAs in each of three widely used sgRNA libraries [10–12] contain at least one common genetic variant (minor allele frequency > 5% in the 1KGP cohort), and > 2% of these sgRNAs contain a variant in the individual human genome of an induced pluripotent stem cell (iPSC) line WTC, commonly used for disease modeling [13] (Fig. 1c, Additional file 1: Figure S1). Failing to account for variants can reduce the efficacy of sgRNAs and also generate unexpected off-target effects [7]. These results emphasize the importance of designing sgRNAs using the personal genome of the patient or cell line where they will be deployed, or at least accounting for both heterozygous and homozygous genetic variants when interpreting the results using sgRNA libraries designed for the reference genome.

Heterozygous genetic variants can be leveraged to establish new therapeutic and research possibilities with allele-specific genome editing. Questions that allele-specific editing could help address include haploinsufficiency, imprinting, and allele-specific gene regulation, as well as discovery and correction of heterozygous disease variants. One promising example is genome surgery to treat dominant negative disease by excising only the disease-causing copy of a gene, an approach which rescues healthy phenotypes in cell and animal models of dominant negative diseases including Huntington’s disease [14] and retinitis pigmentosa [15, 16].

We assessed the strategy of allele-specific gene editing genome wide by identifying pairs of allele-specific sgRNA sites for each human protein-coding gene that could generate a genomic excision and eliminate protein production from just one allele. Given a Cas nuclease, an estimated maximum distance between the two sgRNAs on the haplotype to be excised, and allele-specific sgRNA sites based on the individual’s genetic variants, it is possible to classify genes—or other genomic elements such as enhancers—as putatively targetable or not (Additional file 1: Figure S2). We use the term putatively targetable when a pair of allele-specific sgRNAs exists but has not yet been tested, because it will not always be possible to cut specifically at a site and coding exon excision will not always stop the expression. Previous work indicates that excision of large genomic fragments (> 10 kb) is feasible and that excision of coding exons via sgRNAs targeted to flanking non-coding regions, such as promoter or intronic regions, can mediate gene knockout [17–19].

As an example, suppose we choose a maximum distance of 10 kb between sgRNAs, requiring the sgRNAs to be within the gene including introns, and consider 11 Cas varieties (Additional file 1: Table S1). Then, the average individual from 1KGP is putatively targetable for allele-specific excision at 64% of protein-coding genes [14]. The rate of putatively targetable individuals per gene is evenly distributed across the chromosomes but varies by Cas nuclease and gene (Fig. 2b). For genes that are not putatively targetable, additional allele-specific sgRNA sites may be found by leveraging non-coding variants up- and downstream of the gene, or even in distal enhancers for the gene [14]. As a second example, we found that by simply including the 5-kb flanking regions of each gene, we can increase the mean proportion of putatively targetable protein-coding genes per 1KGP individual to 75%. A caveat to this is that the specificity of each sgRNA pair will vary greatly, potentially even between sgRNAs targeting the same pair of heterozygous variants. Therefore, we conclude that allele-specific excision may be applicable to the vast majority of genes in most human genomes, but extensive experimental optimization for efficiency and specificity will be needed.

Since some genes in a given individual do not have a pair of allele-specific sgRNAs, we asked if gene silencing with a single allele-specific sgRNA within the coding sequence (single-guide strategy) makes more genes putatively targetable. We compared paired-guide and single-guide strategies for allele-specific gene knockout in the individual human genome of the WTC iPSC line [13] and found that more than twice as many genes are putatively targetable with paired guides despite the requirement of two editing sites (Additional file 1: Figure S3). This follows intuition, because one or both sgRNAs can fall in introns or untranslated regions (providing more potential editing sites with dual guides), whereas individual sgRNAs in the single-guide strategy are limited to coding regions. Genes that are putatively targetable with a single- and not paired-guide approach tend to have less than two heterozygous variants in the gene, indicating that a lack of multiple variants is the primary reason a paired-guide strategy fails. These genes could be putatively targetable with a paired-guide strategy by incorporating flanking, promoter, or other regulatory regions. Again, putative editing sites and sgRNAs need to be experimentally validated. We conclude that in most cases, allele-specific gene targeting may be greatly enhanced by including paired guides in the experimental approach.

Genome editing sgRNAs do not need to be designed one genome at a time. Variants that impact sgRNA sites are often shared among large proportions of the individuals within and sometimes between populations due to haplotype structure. Previous work had a similar goal of developing sgRNAs for broad use [6]. However, that work focused on targeting invariant (or low variation) segments of the genome towards homozygous, single-sgRNA-based CRISPR editing while AlleleAnalyzer focuses on taking advantage of genome variation for allele-specific editing with individual sgRNAs, or pairs of sgRNAs. Allele sharing varies by population and locus, as individuals with common ancestry will share haplotypes that harbor specific sets of variants. We therefore developed an algorithm to identify allele-specific sgRNA guide pairs for a given gene that cover the maximum number of individuals in a population; these have the broadest therapeutic potential, similar to designing a drug to treat as many people as possible (Additional file 1: Figure S4). Specifically, our method seeks to cover the most people with the fewest sgRNA pairs using their shared heterozygous variants; this is similar to the “set cover” problem in that the algorithm identifies an optimal combination rather than simply selecting most shared sgRNA pairs, which could disproportionately favor one group over another [20]. Our algorithm generates optimized pairs of sgRNAs that can be used to study or treat genetic diseases in large groups, potentially eliminating the need to develop new sgRNA pairs for each patient or cell line, with practical implications for the development of genome surgery as a field. Our algorithm can also be used to identify sgRNA pair combinations applicable to a custom cohort; this enables researchers to design guides that are maximally shared among multiple cell lines, for example, which would improve the experimental efficiency. Optimized sgRNAs can then be validated for each individual via targeted genotyping, reducing sequencing and sgRNA synthesis costs.

**Fig. 3:**
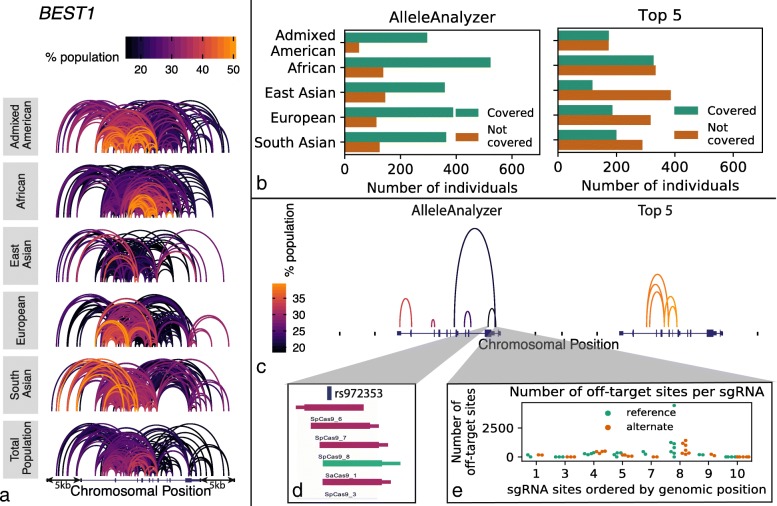
Targeting pairs of allele-specific polymorphisms. **a** Common shared targetable variant pairs for SpCas9 and SaCas9 vary greatly by population, as demonstrated in the gene *BEST1* including the 5-kb flanking regions in the five 1KGP superpopulations. **b** AlleleAnalyzer optimizes sgRNA pair combinations to best cover a cohort, which performs much better compared to the naïve approach of selecting the most highly shared pairs (top 5). **c** The pairs identified by the AlleleAnalyzer and the top 5 approaches demonstrate disparate patterns of sharing among the entire 1KGP population. The height of the arcs is only for visualization purposes and is not otherwise meaningful. **d** AlleleAnalyzer designs sgRNAs, colored by Cas variety, here with SpCas9 represented by purple and SaCas9 by green. **e** For each variant, or sgRNA site, multiple sgRNAs can be designed on both the reference and alternate alleles, depending which is to be targeted. Each sgRNA, then, has its own set of off-target sites, predicted using the incorporation of the CRISPOR tool in AlleleAnalyzer

**Fig. 4:**
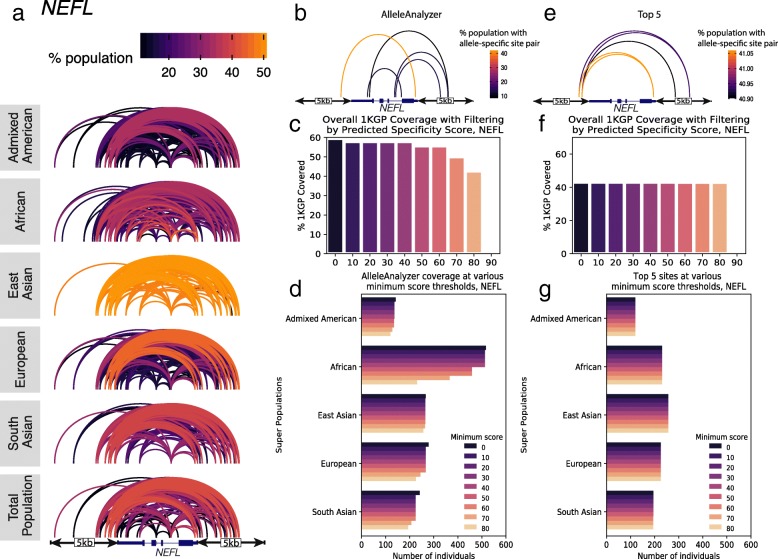
sgRNA pair optimization for coverage of groups. **a** Variant pairs in *NEFL* and the flanking 5 kb that are shared by at least 10% of the 1KGP cohort. These are pairs of variants, not pairs of sgRNAs, so reflect potential dual-guide editing sites prior to designing or filtering sgRNAs. Ten percent was chosen for visualization purposes. **b** Five variant pairs identified by AlleleAnalyzer to achieve greatest possible coverage of the 1KGP cohort. **c** Coverage of the 1KGP cohort with the AlleleAnalyzer set of five pairs at various minimum predicted specificity score thresholds. **d** Coverage of each super population in the 1KGP cohort with the AlleleAnalyzer set of five pairs at various minimum predicted specificity score thresholds. **e** Top 5 shared variant pairs in the 1KGP cohort. **f** Coverage of the 1KGP cohort with the “top 5” set of pairs at various minimum predicted specificity score thresholds. **g** Coverage of each super population in the 1KGP cohort with the “top 5” set of pairs at various minimum predicted specificity score thresholds

As a case study, we investigated the feasibility of excising at least 1 coding exon of *BEST1*, which can cause dominant negative macular degeneration [21]. Considering the gene plus 5 kb of flanking sequence on either side, and allowing 10 kb between each sgRNA in a pair, there are 563 pairs of allele-specific sgRNA sites for SpCas9 that are shared by > 10% of all 1KGP individuals, with the number and composition of these pairs varying across 1KGP populations (Fig. 3a). We sought to identify an optimal combination of 5 allele-specific sgRNA pairs to potentially target the majority of the 1KGP cohort. We found that a combination of 5 allele-specific sgRNA pairs could putatively excise at least 1 coding allele of *BEST1* while leaving the other allele intact in ~ 78% of the overall 1KGP population. This compares to only 48% that would be covered by the naïve approach of selecting a combination of the top 5 most highly shared pairs (Fig. 3b, c). At each sgRNA site, multiple sgRNAs are possible for both the reference and alternate alleles (Fig. 3d) depending on which is being targeted in the research subject. Each of these sgRNAs has a unique off-target profile (Fig. 3e, Additional file 1: Figure S5, Additional file 2: Table S2), which we identified by integrating the tool CRISPOR into AlleleAnalyzer [3]. Previous studies have predicted that genetic variation may have a large impact on the off-target landscape [6, 7]. One of these produced a set of “platinum” sgRNAs for all coding genes identified based on the target sites having low genetic variation and predicted off-targets, including off-targets generated by genetic variation [6]. Using the WTC genome, we compared these sgRNAs to those produced by AlleleAnalyzer in the gene *PCSK9*. We determined that the set of platinum sgRNAs indeed has high predicted sensitivity and specificity in WTC, but some loci lack platinum sgRNAs; AlleleAnalyzer is able to design personalized sgRNAs in these loci, making it a flexible option that we expect will be useful in practice (Additional file 1: Figure S6). CRISPOR specificity scoring will be robust to most variation as it searches for all similar sites in the genome to an sgRNA with up to 4 mismatches. Additionally, the predictive power of these scores is low in general [3]. AlleleAnalyzer does allow the user to filter sgRNAs for predicted specificity, and doing so can impact relative coverage using either the AlleleAnalyzer or the top 5 pair methods, as we demonstrated in 6 therapeutically relevant genes (Fig. 4, Additional file 1: Figure S7–S11). Therefore, particularly in cases of therapeutic development, we recommend rigorous experimental whole-genome off-target analysis. Together, these results demonstrate important considerations for allele-specific sgRNA design.

The bioinformatics methods from this study have been implemented in AlleleAnalyzer, an open-source Python software tool (Additional file 1: Figure S12, S13). This tool designs personalized and allele-specific sgRNAs for unique individuals and cohorts, given their genetic variants, and optimizes sgRNA pairs to cover many individuals based on shared variants. To our knowledge, this is the first computational resource that designs personalized and allele-specific CRISPR sgRNAs. AlleleAnalyzer accounts for single-nucleotide variants and short insertions and deletions, and currently supports 11 Cas proteins while providing user options to add new Cas proteins, thus expanding and building upon the existing repertoire of sgRNA design tools (Additional file 3: Table S3). The AlleleAnalyzer toolkit and tutorials are available along with the database of annotated 1KGP variants at https://github.com/keoughkath/AlleleAnalyzer under the MIT license (DOI: 10.5281/zenodo.3354488

## Conclusions

The genetic-variation-aware sgRNA design tool AlleleAnalyzer is an important step towards effective deployment of CRISPR-based technologies in diverse genomes, including but not limited to research and therapeutic development for once incurable dominant negative diseases.

## Methods

### PAM occurrence in the human reference genome

#### PAM frequency

The AlleleAnalyzer tool includes a script enabling scanning of a reference genome fasta file for existing PAM sites. We used this to identify PAM sites for 11 Cas types (Additional file 1: Table S1) in the reference human genomes hg19 and hg38. These are viewable in publically accessible UCSC Genome Browser sessions (hg19: https://bit.ly/2GB9cXK, hg38: https://bit.ly/2BZAmVh), with a sample view in Additional file 1: Figure S14.

#### PAM size

PAM sizes were equated as the sum of non-N (A, C, G, or T) bases in a PAM site. Thus “NGG” for SpCas9 would have size 2, and “NNGRRT” for SaCas9 would have size 4.

### Analysis of variants in commonly used sgRNA libraries

For each sgRNA library, genomic coordinates for the protospacer regions were obtained from the relevant supporting manuscript. These were converted into BED files including the protospacer and PAM sites. Bcftools [22] was then used to extract the variants with a minor allele frequency (MAF) > 5% from the 1000 Genomes data, or variants from WTC with no MAF restriction. Variants that fell in the “N” position of the PAM were removed.

### AlleleAnalyzer analyses

#### Annotation of variants

Genetic variants were determined to generate or destroy an allele-specific sgRNA site if they were proximal to or in a PAM site (Fig. 1a). Sufficient proximity to a PAM site was defined for this study as 20 bp based on the common length of sgRNA recognition sequences. For all Cas varieties, this was the 20-bp 5′ of the PAM, except for cpf1 (Cas12a) for which it was 3′ of the PAM. The sgRNA design tools that are part of AlleleAnalyzer allow different user-defined sgRNA lengths and addition of Cas enzymes and PAMs. There is evidence to suggest that genetic variants that generate or destroy a PAM are more likely to lead to allele-specific Cas activity compared to those in the seed sequence [1]; AlleleAnalyzer thus provides options to differentiate between CRISPR sites in a PAM site versus the sgRNA recognition sequence. All variants genome wide were annotated for the 1KGP cohort for reference genomes hg19 and hg38; an example subset of these data for the first 100 variants annotated by 1KGP on chromosome 1 in reference genome hg19 is available in Additional file 4: Table S4. All variants in the ExAc dataset were annotated for the reference genome hg19 only, as that dataset is not available in hg38.

#### Generation of gene set

The analyzed gene set was compiled using the canonical transcripts for RefSeq gene annotations for human reference genome hg19 and hg38 downloaded using the UCSC Table Browser [23]. Values reported in the text are for hg19 unless stated otherwise, but 1KGP analyses were conducted for both reference genomes with similar results.

#### Allele-specific putative gene targetability genome wide

Putative allele-specific targetability of a gene is defined here as whether a gene contains a pair of allele-specific sgRNA sites for at least 1 of the 11 Cas enzymes evaluated that are less than 10 kb apart on the same haplotype in an individual that will disrupt a coding exon (Additional file 1: Figure S2). This metric was calculated for each gene for all 2504 1KGP individuals. It was not calculated for the ExAc cohort as that dataset contains only exome rather than whole-genome variants.

#### Set cover analysis

In order to find the optimal set of sgRNAs, we initialized two vectors of indicator variables that are constrained to be binary, one for sgRNAs and one for individuals. When these indicator variables are set to 1, this means a sgRNA is chosen or a person is covered, respectively. We then specified the objective function to maximize the sum of person indicator variables. Next, we set the constraint on maximum value allowed for the sum of sgRNA indicator variables. Finally, we set up the constraints we have deduced from the data, the bipartite graph of sgRNAs and patients targetable by them. This graph gets translated into multiple inequality constraints that specify that if a person indicator is 1, then at least one of its connected sgRNA indicators must also be 1. Having specified all these elements of the problem, we are free to solve it with any number of integer linear programming solvers; we used the Python package PuLP [24]. We then extract the final values of the indicator variables from the solution and have our set of sgRNAs that fulfill the chosen objective. The specific Python implementation of the constraints and objective function and subsequent call to an integer linear programming solver can be seen in the GitHub repository for this tool. This is visualized in Additional file 1: Figure S4.

#### Comparison of AlleleAnalyzer to platinum sgRNAs from Scott and Zhang [6]

Platinum sgRNAs for SpCas9 were obtained from the supplementary materials of their paper [6]. Personalized non-allele-specific sgRNAs were designed for *PCSK9* exon 1 in WTC using AlleleAnalyzer. This analysis was done in reference genome hg19.

### WTC sequencing

The genome for the iPSC line WTC [13] was sequenced by the Allen Institute for Cell Science. Analysis and variant calls in the reference genome hg19 were done according to GATK version 3.7 best practices [25] and phased using Beagle version 4.1 with default settings [26].

### WTC targetability analysis

Variant annotation procedures were the same as in the 1KGP analysis and ExAc.

### Packages used

#### Python

Docopt was used for handling of command-line arguments. Pandas [27] version 0.21.0, NumPy [28] version 1.13.3, and elements of the standard Python distribution sys, os, and regex were used for multiple aspects of data analysis. PuLP [24] version 1.6.8 was used for set cover analysis. PyTables [29] was used for data management. Biopython [30] and pyfaidx [31] were used for fasta processing. Scripts from CRISPOR [3] were integrated into AlleleAnalyzer to facilitate specificity scoring of sgRNAs. Seaborn [32], matplotlib [33] and PyUpset [34] were used for plotting.

#### R

Packages used to generate arcplots included viridis version 0.5.1, viridisLite version 0.3.0, igraph version 1.1.2, ggraph version 1.0.0, ggplot2 version 2.2.1, reshape2 version 1.4.3, dplyr version 0.7.4, tidyr version 0.7.2, and readr version 1.1.1.

#### Bioinformatics

Bcftools version 1.9 was used to manipulate VCF and BCF files.

### Code availability and scripts

All data processing and analysis scripts as well as the sgRNA design tool are located at github.com/keoughkath/AlleleAnalyzer, available under the MIT license (DOI: 10.5281/zenodo.3354488). Scripts were written in Python version 3.6.1, R version 3.3.2, and Bash version 3.2.57.

## Additional files

Additional file 1: Supplementary Figure S1-S14 and supplementary **Table S1.** (DOCX 12973 kb)

Additional file 2: **Table S2.** AlleleAnalyzer guides for SpCas9 and SaCas9 in *BEST1* for optimal coverage of the 1000 genomes cohort, as shown in Fig. 3b-e. Guides with “---” as sequence indicate loss of PAM site due to a variant, and therefore non-targetability of that allele. (XLSX 13 kb)

Additional file 3: **Table S3.** Comparison of AlleleAnalyzer features with other commonly used CRISPR sgRNA design tools. (XLSX 12 kb)

Additional file 4: **Table S4.** PAM site annotation example set for the first 100 variants from the 1KGP on chromosome 1. Full dataset is available as denoted in the data availability section. (XLSX 21 kb)

## Data Availability

1KGP phase 3 data were downloaded from the 1KGP website (http://www.internationalgenome.org/). ExAc data were downloaded from the ExAc website (http://exac.broadinstitute.org/). The reference hg19 and hg38 genome data were downloaded from the UCSC genome browser. The 1KGP and ExAc analysis datasets have been made available for public access online at UCSF Dash (https://datashare.ucsf.edu/stash/dataset/doi:10.7272/Q63F4MSR). Additionally, PAM sites identified in reference genomes hg19 and hg38 are viewable in UCSC Browser sessions (hg19: https://bit.ly/2GB9cXK or https://genome.ucsc.edu/cgi-bin/hgTracks?db=hg19&lastVirtModeType=default&lastVirtModeExtraState=&virtModeType=default&virtMode=0&nonVirtPosition=&position=chr11%3A61717368%2D61717468&hgsid=743058527_XLIEJrwnSVsZQLgeXUfU7NKQWeNn; hg38: https://bit.ly/2BZAmVh or https://genome.ucsc.edu/cgi-bin/hgTracks?db=hg38&lastVirtModeType=default&lastVirtModeExtraState=&virtModeType=default&virtMode=0&nonVirtPosition=&position=chr11%3A61957117-61957165&hgsid=710108079_SecTcyDrgBPU4AocIPTRF2Uq4Omd). WTC whole-genome sequencing data is made available by the Allen Institute at https://www.allencell.org/genomics.html. In addition to the GitHub repository for AlleleAnalyzer (github.com/keoughkath/AlleleAnalyzer, available under the MIT license) [35], an archived release of the software is available under DOI:10.5281/zenodo.3354488 provided through Zenodo.
