# Supplementary material for: AlleleAnalyzer: a tool for personalized and allele-specific sgRNA design
Source: Genome Biol. 2019 Aug 15;20:167. doi: 10.1186/s13059-019-1783-3 (PMC6694686; doi:10.1186/s13059-019-1783-3)
Supplement: Supplementary file 1 — Supplementary Figure S1-S14 and supplementary Table S1. (DOCX 12973 kb) [file 13059_2019_1783_MOESM1_ESM.docx]

**Fig. S1** WTC variants in widely used sgRNA libraries.

## ****

## **Fig. S2** A pair of allele-specific sgRNA sites is defined at putatively targetable if their predicted excision will disrupt at least one protein-coding exon

## ****

## **Fig. S3** In this Upset plot, each filled in circle indicates which set, named on the left, is being shown in the above barplot [34]. A line between filled-in circles denotes the intersection of these sets, similar to a Venn diagram, and the size of this intersection is then shown by the above barplot. The barplot on the left shows the total size of each individual named set. Many more genes are targetable in the genome of WTC with a paired (dual)- as opposed to single-guide strategy. The number of variants in a gene is influential in determining targetability. Many genes that are not dual- or single-guide targetable have very few variants, and the genes that are only targetable with a single-guide approach compared to a dual-guide approach also tend to have fewer variants. All 11 Cas varieties are considered in this analysis.

**Fig. S4** Overview of the AlleleAnalyzer set cover approach to maximize population coverage by identifying optimal variant pair combinations for sgRNA design.

**Fig. S5** CRISPOR specificity scores for AlleleAnalyzer sgRNAs from Figure 3d.

**Fig. S6** IGV screenshots comparing platinum sgRNAs from Scott & Zhang 2017 to those designed with AlleleAnalyzer for WTC. All sgRNAs shown are for SpCas9. A) Platinum sgRNAs in the gene *PCSK9*. No platinum sgRNAs are available for the first exon, the most likely to produce a gene knockout, because that exon is prone to genetic variation. B) WTC has 3 heterozygous variants in exon 1 of *PCSK9,* indicated by the red and blue line in the “WTC variants” track. AlleleAnalyzer designs personalized, non-allele-specific (genetic-variant-aware) sgRNAs that avoid these heterozygous variants, and therefore is able to target this exon in WTC.

**Fig. S7** A) Variant pairs in *BEST1* and the flanking 5kb that are shared by at least 20% of the 1KGP cohort. These are pairs of variants, not pairs of sgRNAs, so reflect potential dual-guide editing sites prior to designing or filtering sgRNAs. 20% was chosen for visualization purposes. B.) 5 variant pairs identified by AlleleAnalyzer to achieve greatest possible coverage of the 1KGP cohort. C.) Coverage of the 1KGP cohort with the AlleleAnalyzer set of 5 pairs at various minimum predicted specificity score thresholds. D.) Coverage of each super population in the 1KGP cohort with the AlleleAnalyzer set of 5 pairs at various minimum predicted specificity score thresholds. E.) 5 top shared variant pairs in the 1KGP cohort. F.) Coverage of the 1KGP cohort with the “Top 5” set of pairs at various minimum predicted specificity score thresholds. G.) Coverage of each super population in the 1KGP cohort with the “Top 5” set of pairs at various minimum predicted specificity score thresholds.

**Fig. S8** A) Variant pairs in *HSPB1* and the flanking 5kb that are shared by at least 20% of the 1KGP cohort. These are pairs of variants, not pairs of sgRNAs, so reflect potential dual-guide editing sites prior to designing or filtering sgRNAs. 20% was chosen for visualization purposes. B.) 5 variant pairs identified by AlleleAnalyzer to achieve greatest possible coverage of the 1KGP cohort. C.) Coverage of the 1KGP cohort with the AlleleAnalyzer set of 5 pairs at various minimum predicted specificity score thresholds. D.) Coverage of each super population in the 1KGP cohort with the AlleleAnalyzer set of 5 pairs at various minimum predicted specificity score thresholds. E.) 5 top shared variant pairs in the 1KGP cohort. F.) Coverage of the 1KGP cohort with the “Top 5” set of pairs at various minimum predicted specificity score thresholds. G.) Coverage of each super population in the 1KGP cohort with the “Top 5” set of pairs at various minimum predicted specificity score thresholds.

**Fig. S9** A) Variant pairs in *MFN2* and the flanking 5kb that are shared the 1KGP cohort. These are pairs of variants, not pairs of sgRNAs, so reflect potential dual-guide editing sites prior to designing or filtering sgRNAs. B.) 5 variant pairs identified by AlleleAnalyzer to achieve greatest possible coverage of the 1KGP cohort. C.) Coverage of the 1KGP cohort with the AlleleAnalyzer set of 5 pairs at various minimum predicted specificity score thresholds. D.) Coverage of each super population in the 1KGP cohort with the AlleleAnalyzer set of 5 pairs at various minimum predicted specificity score thresholds. E.) 5 top shared variant pairs in the 1KGP cohort. F.) Coverage of the 1KGP cohort with the “Top 5” set of pairs at various minimum predicted specificity score thresholds. G.) Coverage of each super population in the 1KGP cohort with the “Top 5” set of pairs at various minimum predicted specificity score thresholds.

**Fig. S10** A.) Variant pairs *PSCK9* and the flanking 5kb that are shared by at least 15% of the 1KGP cohort. These are pairs of variants, not pairs of sgRNAs, so reflect potential dual-guide editing sites prior to designing or filtering sgRNAs. 15% was chosen for visualization purposes. B.) 5 variant pairs identified by AlleleAnalyzer to achieve greatest possible coverage of the 1KGP cohort. C.) Coverage of the 1KGP cohort with the AlleleAnalyzer set of 5 pairs at various minimum predicted specificity score thresholds. D.) Coverage of each super population in the 1KGP cohort with the AlleleAnalyzer set of 5 pairs at various minimum predicted specificity score thresholds. E.) 5 top shared variant pairs in the 1KGP cohort. F.) Coverage of the 1KGP cohort with the “Top 5” set of pairs at various minimum predicted specificity score thresholds. G.) Coverage of each super population in the 1KGP cohort with the “Top 5” set of pairs at various minimum predicted specificity score thresholds.

**Fig. S11** A.) Variant pairs in *RHO* and the flanking 5kb that are shared by at least 10% of the 1KGP cohort. These are pairs of variants, not pairs of sgRNAs, so reflect potential dual-guide editing sites prior to designing or filtering sgRNAs. 10% was chosen for visualization purposes. B.) 5 variant pairs identified by AlleleAnalyzer to achieve greatest possible coverage of the 1KGP cohort. C.) Coverage of the 1KGP cohort with the AlleleAnalyzer set of 5 pairs at various minimum predicted specificity score thresholds. D.) Coverage of each super population in the 1KGP cohort with the AlleleAnalyzer set of 5 pairs at various minimum predicted specificity score thresholds. E.) 5 top shared variant pairs in the 1KGP cohort. F.) Coverage of the 1KGP cohort with the “Top 5” set of pairs at various minimum predicted specificity score thresholds. G.) Coverage of each super population in the 1KGP cohort with the “Top 5” set of pairs at various minimum predicted specificity score thresholds.

## ****

## **Fig. S12** Integrative Genomics Viewer (IGV) track view of allele-specific (upper track) and non-allele-specific personalized (middle track) sgRNAs designed with AlleleAnalyzer for SpCas9, SaCas9 and cpf1 (Cas12a) in and near the first exon of *BEST1* in WTC. Colors indicate the type of Cas protein, lavender for SpCas9, blue for SaCas9, and orange for cpf1 (Cas12a). Allele-specific guides are shaded according to position of the variant in the guide, with variants closer to the PAM being darker based on their putative greater specificity. The track labeled “WTC genetic variants” (top) denotes genetic variants in WTC in this locus, where a solid color indicates a homozygous variant, while red and blue indicate a heterozygous variant. The bottom track shows the RefSeq annotation for the first exon of this gene, *BEST1*, in hg38. sgRNA labels include type of Cas protein, unique identification number, and position of the variant (if the sgRNA contains a variant) in the sgRNA relative to the start of the PAM sequence. Due to space constraints based on the number of sgRNAs being displayed, the non-allele-specific personalized sgRNAs are shown in a compressed format relative to the allele-specific sgRNAs. This is a feature of the IGV intended to improve visualization.

## ****

## **Fig. S13** Flowchart for the AlleleAnalyzer software tool.

**Fig. S14** Screenshot of UCSC Browser Tracks of PAM sites identified by AlleleAnalyzer.

| **Common name(s)** | **Abbreviation** | **PAM** | **Properties** |
| --- | --- | --- | --- |
| SpCas9 | SpCas9 | NGG | *Streptococcus pyogenes* (Sp) Cas9., most widely used version with dozens of variants using same PAM, e.g. eSpCas9, SpCas9-HF1, eSpCas9 1.1 and more (Jinek et al. 2012) |
| SpCas9 VRER Variant | SpCas9-V1 | NGCG | Version of  SpCas9 with alternative targeting range (Kleinstiver et al. 2015) |
| SpCas9 EQR Variant | SpCas9-V2 | NGAG | Version of  SpCas9 with alternative targeting range (Kleinstiver et al. 2015) |
| SpCas9 VQR Variant | SpCas9-V3 | NGAN or NGNG | Version of  SpCas9 with wider targeting range (Kleinstiver et al. 2015) |
| SaCas9 | SaCas9 | NNGRRT | *Staphylococcus aureus* (Sa) Cas9.  Small relative to SpCas9, (Horvath et al. 2008, Jiang et al. 2013) |
| SaCas9 KKH Variant | SaCas9-V1 | NNNRRT | Version of SaCas9 with 2 to 4-fold increased targeting range relative of SaCas9 (Kleinstiver et al. 2015) |
| nmCas9 | nmCas9 | NNNNGATT | *Neisseria meningitidis* (Nm) Cas9, with different PAM site (Hou et al. 2013) |
| cpf1, Cas12a | cpf1 | TTTN | Multiple variations, notably opposite orientation system and sticky-end cut rather than blunt. Multiple species exist, including from *Acidaminucoccus* and *Lachnospiraceae.* (Zetsche et al. 2015) |
| StCas9 1 | StCas9-V1 | NNAGAA | *Streptococcus thermophilus* (St) Cas9. Smaller relative of SpCas9. Increased specificity. (Kleinstiver et al. 2015, Muller et al. 2016) |
| StCas9 2 | StCas9-V2 | NGGNG | *Streptococcus thermophilus* (St) Cas9. Smaller relative of SpCas9. Increased specificity. (Muller et al 2016) |
| cjCas9 | cjCas9 | NNNNACA | *Campylobacter jejuni* Cas9. Smallest Cas9 ortholog to date, easy to package (Kim et al. 2017) |

**Table S1** 11 types of Cas enzyme were evaluated, each of which has a distinct PAM site.
